# Photooxidation of the Phenolate Anion is Accelerated at the Water/Air Interface

**DOI:** 10.1021/jacs.2c04935

**Published:** 2022-07-28

**Authors:** Caleb J. C. Jordan, Eleanor A. Lowe, Jan R. R. Verlet

**Affiliations:** Department of Chemistry, Durham University, Durham, DH1 3LE, United Kingdom

## Abstract

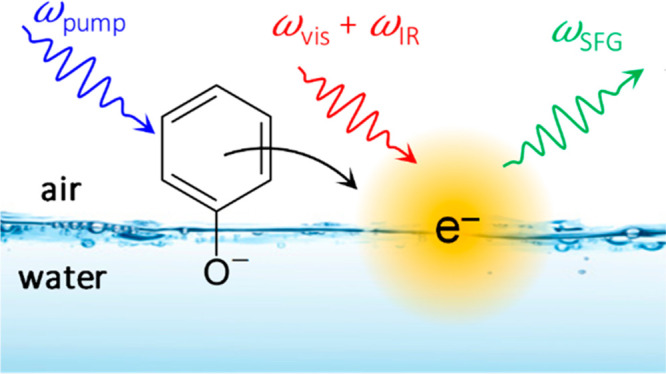

Molecular photodynamics can be dramatically affected at the water/air interface. Probing such dynamics is challenging, with product formation often probed indirectly through its interaction with interfacial water molecules using time-resolved and phase-sensitive vibrational sum-frequency generation (SFG). Here, the photoproduct formation of the phenolate anion at the water/air interface is probed directly using time-resolved electronic SFG and compared to transient absorption spectra in bulk water. The mechanisms are broadly similar, but 2 to 4 times faster at the surface. An additional decay is observed at the surface which can be assigned to either diffusion of hydrated electrons from the surface into the bulk or due to increased geminate recombination at the surface. These overall results are in stark contrast to phenol, where dynamics were observed to be 10^4^ times faster and for which the hydrated electron was also a photoproduct. Our attempt to probe phenol showed no electron signal at the interface.

Chemistry at aqueous interfaces can be quite different from their bulk counterparts^1^ with different kinetics or new reaction pathways.^2−5^ Photochemistry of molecules may be particularly sensitive to their environment because electronically excited states and their dynamics depend critically on changes to potential energy surfaces.^6−11^ Probing photodynamics at aqueous interfaces is a major scientific goal, with particular atmospheric relevance.^3,12^ However, experimentally probing such dynamics has been challenging. Tahara and co-workers recently demonstrated that the photochemistry of aqueous phenol is accelerated by a factor of 10^4^ at the water/air interface^13^ compared to bulk or the gas phase.^14−16^ Their data suggested ultrafast (<100 fs) electron ejection from phenol to form a hydrated electron, e^–^_(aq)_, which then migrated into the bulk within 300 fs, as well as the formation of a hydronium cation at the interfacial layer. Their work probed the products (e^–^_(aq)_, hydronium cation, phenoxyl radical) through their interaction with interfacial water molecules. Specifically, time-resolved heterodyne-detected vibrational sum-frequency generation (SFG) was used to measure transient changes in the interfacial water IR spectrum.^13^ While elegant, such an approach offers a rather indirect probe of the products. It would instead be desirable to probe a product *directly*. This can be done using electronic SFG, where one of the driving and/or SFG fields is resonant with a product.^17,18^ Such electronic SFG is complementary if not more suitable than vibrational SFG.^19,20^

We focus on the phenolate anion at the water/air interface, in part because the bulk spectroscopy (transient absorption) is simpler than that of aqueous phenol,^21,22^ thus facilitating comparison between the surface and bulk. The phenolate anion was excited at 257 nm and probed surface-selectively using time-resolved optically Kerr-gated (OKG) electronic SFG.^23^ The two probe fields were at 720 and 1028 nm, where the former matches the peak absorption of the p ← s transition of e^–^_(aq)_.^24,25^ The resultant SFG field was at 423 nm (see Supporting Information for experimental details). Given the three relevant SFG fields and the absorption spectra of the potential products (e^–^_(aq)_^24,25^ and phenoxyl radical^21,26^), only e^–^_(aq)_ will lead to resonance enhancement at the interface. Therefore, the SFG signal offers a direct measure of the surface concentration of electrons at the water/air interface. The addition of OKG is essential to suppress bulk fluorescence (see Supporting Information).

The square-root of the SFG signal is directly proportional to the surface concentration, *N*_surf_,^27,28^ and thus comparable to transient absorption spectroscopy that probes the bulk. Figure 1 shows the kinetics of the electron concentration at both the surface and bulk and reveals subtle differences. In the bulk, a transient absorption at 720 nm rises to a maximum within 2 ps and then decays to leave an offset by ∼100 ps, in agreement with previous studies.^21,22^ A kinetic model originally used to describe the charge-transfer-to-solvent (CTTS) from aqueous chloride was used to model the signal, which is based on the reaction scheme for the bulk shown in Figure 2.^29^

**Figure 1:**
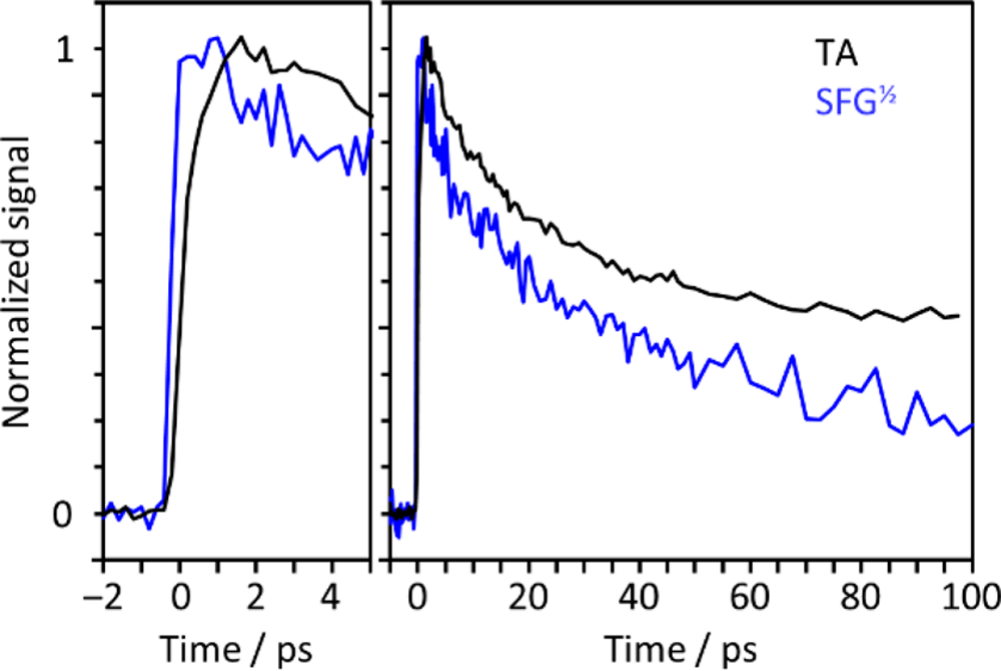
Comparison of bulk transient absorption (black) and electronic SFG (blue), probing e^–^_(aq)_ or [Ph:e^–^]_(aq)_ following excitation of phenolate at 257 nm.

**Figure 2:**
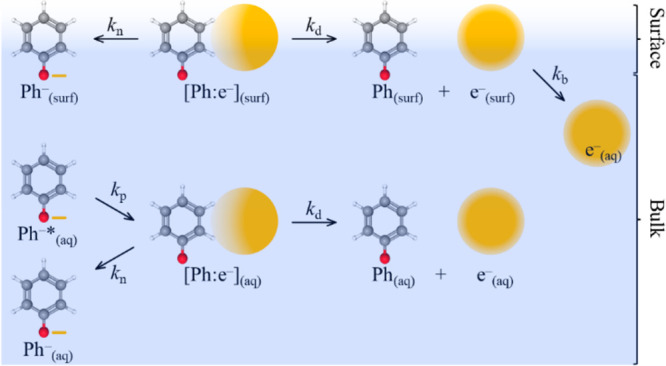
Schematic of kinetic models following photo-oxidation from phenolate at the water/air surface (surf) and in aqueous solution (aq). Phenolate, Ph^–^, is photoexcited, Ph^–^*, and forms a contact pair [Ph:e^–^] with a rate coefficient *k*_p_. [Ph:e^–^] can either dissociate to the phenoxyl radical, Ph, and e^–^, with a rate coefficient *k*_d_ or undergo geminate recombination to reform Ph^–^ with a rate coefficient *k*_n_. The e^–^_(surf)_ can additionally diffuse into the bulk with a rate coefficient *k*_b_. At the surface, *k*_p_ has been omitted for clarity.

In Figure 2, Ph^–^*_(aq)_ represents the photoexcited phenolate anion. An electron is then ejected from Ph^–^*_(aq)_ with a rate coefficient *k*_p_ to form a contact pair, [Ph:e^–^]_(aq)_, in which the phenoxyl radical and electron are combinedly solvated. The contact pair can undergo nonadiabatic recombination to reform Ph^–^_(aq)_ with a rate coefficient *k*_n_, or it can dissociate to form individually solvated Ph_(aq)_ and e_(aq)_^–^ with a rate coefficient *k*_d_. Assuming that the absorption spectrum of [Ph:e^–^]_(aq)_ and e^–^_(aq)_ are indistinguishable, then the total signal is the sum of both these contributions, with the escape yield of e_(aq)_^–^ equating to the long-time (100 ps) offset observed in Figure 1. The relevant rate coefficients (expressed as lifetimes, *k*^–1^) are given in Table 1 and are in good agreement with pervious measurements.^22^

**Table 1:** Lifetimes for Kinetic Processes Following Photo-oxidation of Aqueous Phenolate in the Bulk and at the Water/Air Interface

	*k*_p_^–1^/ps	*k*_n_^–1^/ps	*k*_d_^–1^/ps	*k*_b_^–1^/ps
Bulk	0.5 ± 0.1	32 ± 2	43 ± 4	–
Surface	≪0.2^a^	16 ± 1	11 ± 2	78 ± 6

a Lifetime was actually not included in the fit, but is taken to be (*k*_p_ ≫ *k*_d_ and *k*_n_).

The transient SFG data in comparison show a faster initial rise but subsequently has broadly similar kinetics, suggesting that a similar overall model may apply to the interface. However, rather than reaching a long-term offset, the SFG signal continues to decay on a longer time scale. Thus, there are also clear differences and these must be associated with the differing solvation environments. In the first instance, we attempted to fit the kinetics with the same model. However, from Figure 1, *k*_p_ is clearly much larger than *k*_n_ and *k*_d_ so that the bulk kinetic model can be simplified to
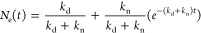
The fit of the SFG data to this model is shown in Figure 3 and shows some clear deviations that the model cannot account for: overshooting the data at early times, then undershooting, and overshooting again at long time. A plot of ln[*N*_e_(*t*)] confirms that the signal is not a simple monoexponential decay (see Supporting Information). Other than the initial rise (associated with *k*_p_), the long-time decay observed is the most obvious difference between the surface and bulk. Bradforth and co-workers accounted for a longer-time (100s ps) decay of e^–^_(aq)_ following CTTS of bulk iodide by including the diffusion of the free e^–^_(aq)_ and I_(aq)_, which can then recombine to form the contact pair [I:e^–^]_(aq)_ and undergo the subsequent competing kinetics in the model.^30^ In the context of phenolate, this would correspond to recombination of e^–^_(aq)_ and Ph_(aq)_ to reform [Ph:e^–^]_(aq)_. On the surface, diffusion can take a different form: diffusion away from the surface would lead to a depletion in the observable SFG signal, and diffusion in two dimensions may have very different kinetics to regenerate [Ph:e^–^]_(surf)_. Both would increase the rate of electron loss at long times. Given the potential complexity, we have accounted for these processes by adding a single exponential decay, noting that diffusion is not necessarily a simple first-order process. The migration of the surface electron to the bulk is shown schematically in Figure 2. The surface electron loss is quantified by the rate coefficient *k*_b_. The overall concentration of electrons at the surface, *N*_surf_(*t*), then becomes (assuming that *k*_p_ ≫ *k*_n_ and *k*_d_, and that the absorption spectrum, or more precisely the χ^(2)^ spectrum, of [Ph:e^–^]_(surf)_ and e^–^_(surf)_ are indistinguishable):
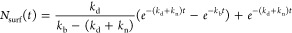


**Figure 3:**
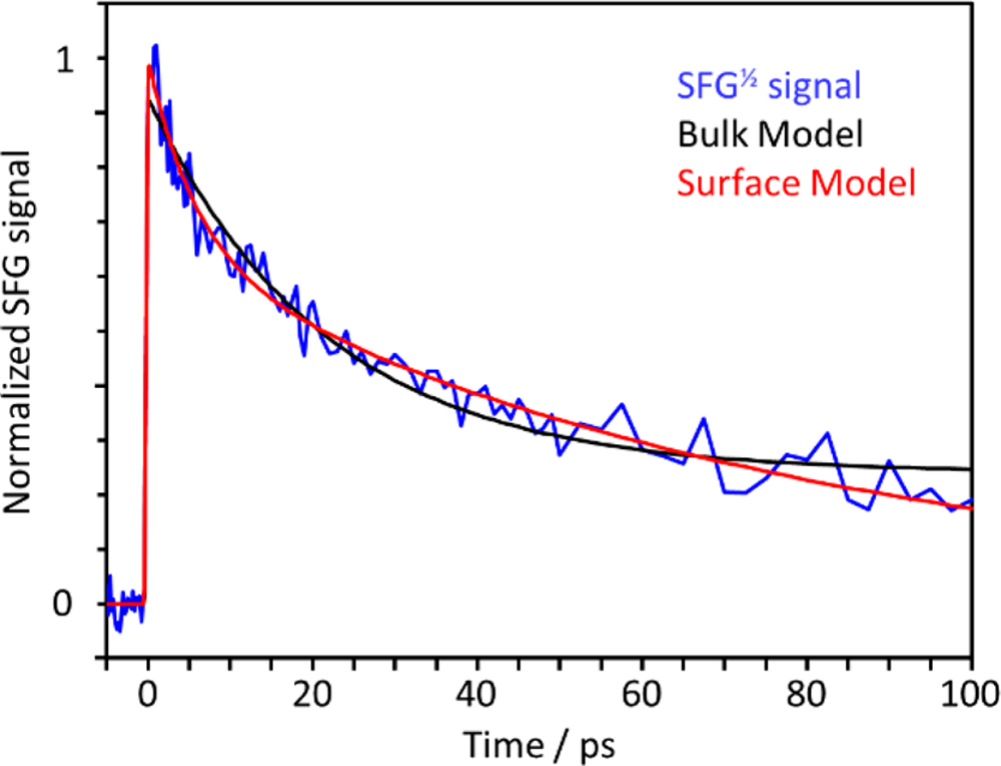
Comparison of surface signal with models. Transient electronic SFG signal (blue), compared to a bulk model (black) and a surface model (red), where the latter accounts for diffusion from the surface or along the surface.

Figure 3 shows the fit of the SFG data to this extended model, which appears significantly improved and captures much of the previous shortcomings. The lifetimes associated with the three processes are given in Table 1. We conclude that the processes taking place at the interface are broadly similar to those in the bulk, but with different rates and with the additional electron loss component at longer times.

All processes (*k*_p_, *k*_d_, and *k*_n_) are faster at the interface. There are several factors that can impact the observed increase in rates. The lower density will affect polarization, electrostatic interactions, hydrogen bonding, and anisotropy of the interactions. Intuitively, the increase in *k*_d_ could be rationalized as the lower density of water molecules would be expected to enable the contact pair [Ph:e^–^]_(surf)_ to dissociate more readily (i.e., larger *k*_d_), because it would be easier to break the solvation cage surrounding both species. The faster nonadiabatic recombination (*k*_n_) could be justified as the free-energy surfaces are altered leading to changes in charge-transfer rate in a Marcus picture. Similar arguments were made to explain the moderate increase in rates observed for aqueous iodide at the interface (increase by a factor of 1.3 in *k*_d_ and 1.4 in *k*_n_).^20^ The larger effect for phenolate compared to iodide may be associated with the system’s size or with other factors such as electric fields at the surface and the dipole moment of the phenoxyl radical, which points toward the vapor phase and may direct the initial charge transfer forming the contact pair.

The other clear difference between the surface and the bulk signals is an almost instantaneous appearance of the signal (*k*_p_^–1^ ≪ 200 fs, limited by our time resolution). The transient absorption at 720 nm corresponds to the fully thermalized [Ph:e^–^]_(surf)_, but this is preceded by a transient which initially starts in the IR and red-shifts as the contact pair becomes solvated.^31^ In the SFG experiment, the 1028 nm field will be resonant with this presolvated contact pair at earlier times. Additionally, the larger spatial distribution of the presolvated electron is likely associated with a larger χ^(2)^.^32^ Hence, the appearance dynamics are expected to be faster in the SFG experiment compared to the transient absorption, as observed.

Finally, the term *k*_b_, whether associated with the evolution of e^–^_(surf)_ into e^–^_(aq)_ or geminate recombination, is a purely surface effect. Note, e^–^_(aq)_ is also generated in the bulk and could diffuse from the bulk to the surface, although we also note the concentration gradient opposes this. Theory suggests that electron diffusion from the surface to the bulk takes place on an ∼10 ps time scale.^33,34^ This is broadly consistent with *k*_b_ observed here. If geminate recombination contributed to *k*_b_, then the internalization would be even slower suggesting that migration of e^–^_(surf)_ to the bulk may be the dominant process.

While the photo-oxidation of phenolate at the water/air interface differs from the bulk, the difference is modest in comparison to that observed for phenol using time-resolved heterodyne-detected vibrational SFG, where electrons were formed within 100 fs and migrated to the bulk within 300 fs (i.e., before any dynamics associated with *k*_d_).^13^ However, the photodynamical mechanisms are also very different for phenol and phenolate. The decay of phenol involves H atom tunnelling through a barrier connecting the initially excited ^1^ππ* state to the dissociative ^1^πσ* state.^35,36^ At the water/air interface, it was argued that the ^1^πσ* state was stabilized relative to the ^1^ππ* state, with calculations supporting this suggestion.^6,37^ In phenolate, there is no ^1^πσ* state and the overall charge-transfer dynamics differ substantially.^38^ Hence, it is not surprising that the water/air interface might impact the overall dynamics so differently for phenol and phenolate.

Nevertheless, as we are sensitive to e^–^_(surf)_, we have also performed experiments on phenol at the water/air interface (see Supporting Information). However, we observe no signal—i.e., there were no discernible transient changes in SFG over the first few 100 ps, including at *t* ≈ 0. So how can we reconcile the lack of electron signal for phenol? Excitation at 257 nm compared to 266 nm could have an impact and may alter the dynamics,^22,38^ although it would seem likely that hydrated electrons would be formed also at 257 nm. The sensitivity of the phenol experiment may also differ from that of phenolate because of differences in their respective electronic structure (see Supporting Information).^38^ Alternatively, one could ask if the OH stretching region of water is sufficiently specific to identify the various intermediates that could be formed. Regardless, there are clearly very interesting dynamics taking place at the water/air interface with pertinence in many areas of science. We hope our work here will encourage more experiments that probe the products directly.

## Supporting Information Available

Details of the experimental arrangement of both transient absorption and SFG experiments; description of the fluorescence removal; signal processing; and full transient absorption spectra; error analysis; phenol SFG data and consideration of sensitivity. (PDF)
