# Supplementary material for: Photooxidation of the Phenolate Anion is Accelerated at the Water/Air Interface
Source: J Am Chem Soc. 2022 Jul 28;144(31):14012–5. doi: 10.1021/jacs.2c04935 (PMC9376918; doi:10.1021/jacs.2c04935)
Supplement: Supplementary file 1 — ja2c04935_si_001.pdf [file ja2c04935_si_001.pdf]

Supporting Information for

Photooxidation of the Phenolate Anion is Accelerated at the Water/Air Interface

Caleb J. C. Jordan, Eleanor A. Lowe and Jan R. R. Verlet*

Department of Chemistry, Durham University, Durham, DH1 3LE, United Kingdom

Methods

A detailed description of the experimental sum frequency generation (SFG) setup has been published.[1] Time-resolved optical Kerr gated (OKG) electronic SFG used 250 fs pulses at 1028 nm and 60 kHz derived from a commercial laser system (Carbide, Light Conversion). One part provided 1028 nm pulses for the SFG probe. A second part pumped an optical parametric amplifier (OPA) (Orpheus, Light Conversion) producing light at 720 nm as the other SFG driving field. The resultant SFG at 423 nm was collected in the reflection geometry onto a photomultiplier tube and detected via a photon counting circuit. The detected light was spectrally filtered using a combination of bandpass filters to give an effective transmission window of approximately 415 to 430 nm. Pump pulses were produced by 4th harmonic generation of a third part of the 1028 nm light producing 257 nm pump pulses. These were chopped at 30 kHz to enable shot-to-shot background subtraction in measurements. Fluorescence generated by the pump was spectrally filtered and the OKG (benzene) was driven by the residual unused 1028 nm light used to pump the OPA. The SFG experiment was performed in the PPP polarization configuration.

Transient absorption spectra were collected using a home-built spectrometer using the same laser system and has also been detailed previously.[2] The pump is generated in the same manner as for the SFG experiment, while the probe was generated by focussing part of the 1028 nm light into a slowly translating sapphire crystal. The probe continuum spans ~450-950 nm.

For both measurements, the solutions used were 100 mM aqueous sodium phenolate, at pH 11, made by dissolving phenol in aqueous sodium hydroxide solution. All chemicals were from Sigma, used as supplied. High purity water (Millipore, Milli-Q Gradient A10, 18.2 MΩ) was used in all cases. At 100 mM, the surface coverage of phenolate is approximately 7%.[3]

For the phenol experiments, solutions with concentrations at 25, 57 and 200 mM were used, offering surface coverages of between 25, 50 and 80%, respectively. These were chosen to match the conditions of Tahara and coworkers.[4]

Signal analysis

The OKG still allows some fluorescence to pass, which can be separately measured with both probe fields absent. This signal effectively corresponds to a time-resolved OKG fluorescence measurement and is consistent with previous reports: the signal has a fast rise (limited by the OKG response), an initial (~15 ps lifetime) decay, and a large constant offset because the lifetime of fluorescence is on the order of nanoseconds (see Fig. S1). Subtraction of the fluorescence from the total time-resolved signal leaves only the desired electronic SFG signal.

The raw photon counted signal containing SFG light and fluorescence at 423 nm generated by pump excitation of phenolate at 257 nm is shown in Figure S1. Absence of probe light yields the fluorescence signal, which is fitted and subtracted from the overall signal. Each data point in Figure S1 is the product of 1.5×10^4 laser shots. Data in Figure 1 of main text is an average of 7 scans.

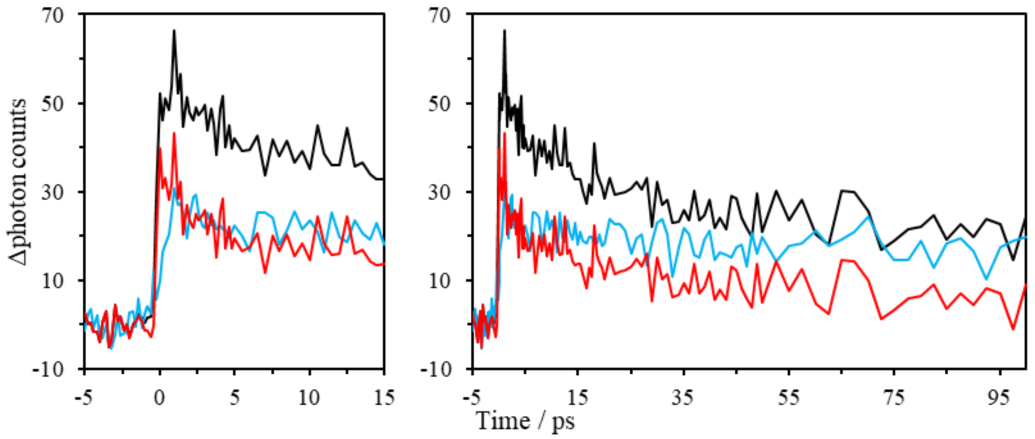

Figure S1: Difference photon counts collected at 423 nm, between pump-on and pump-off channels. Raw scan (black), pump-only (blue) and fluorescence-subtracted (red). The raw and fluorescence subtracted data are from the same scan.

The fluorescence contribution is subtracted by fitting the average of several pump-only scans, shown in Figure S2. The fitting model need only be qualitative, and is taken to be:

$$I_F = A\,e^{-\frac{t}{\tau_F}} + C\,,$$

convoluted with an instrument response function, assumed to be Gaussian and limited by the response of the Kerr medium. The initial decay in the fluorescence, τ_F, is determined to be approximately 15 ± 3 ps in agreement with previously reported kinetics for the fluorescence.[5]

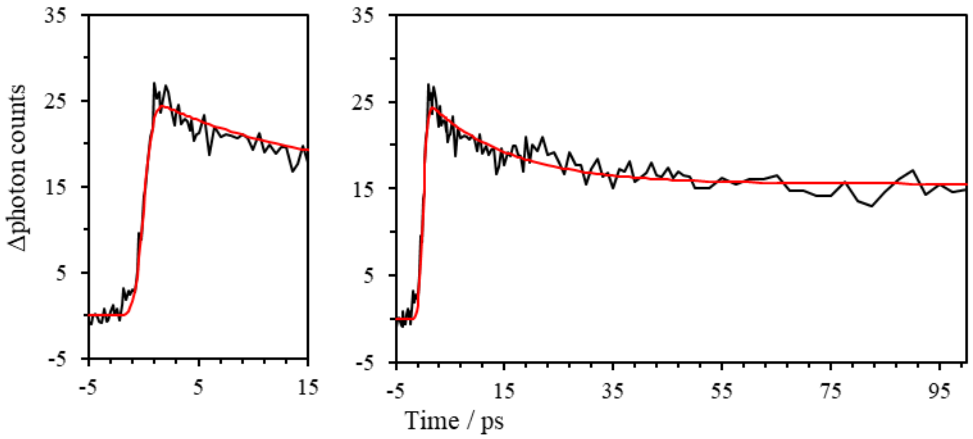

Figure S2: Pump-only signal showing the Kerr-gated fluorescence.

The data shown in the paper is derived from the subtraction in Figure S3

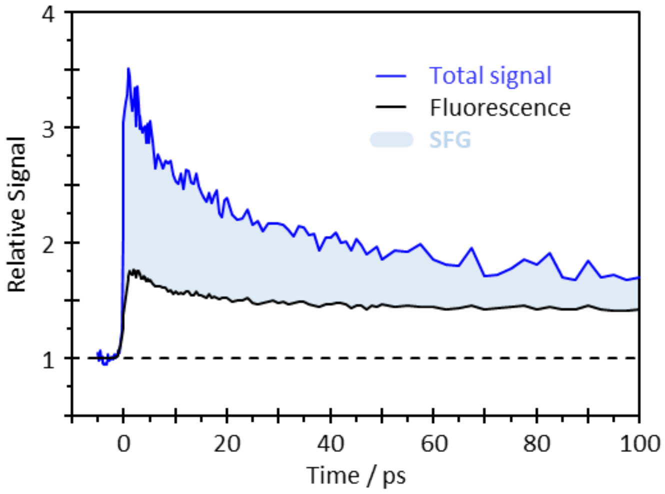

Figure S3: Raw averaged data used to obtain the SFG signal shown in manuscript for phenolate. The blue line is the total signal, the black the fluorescence and the shaded area is the SFG signal, which is then square-rooted in Figure 1 of main text.

Transient absorption (TA) data

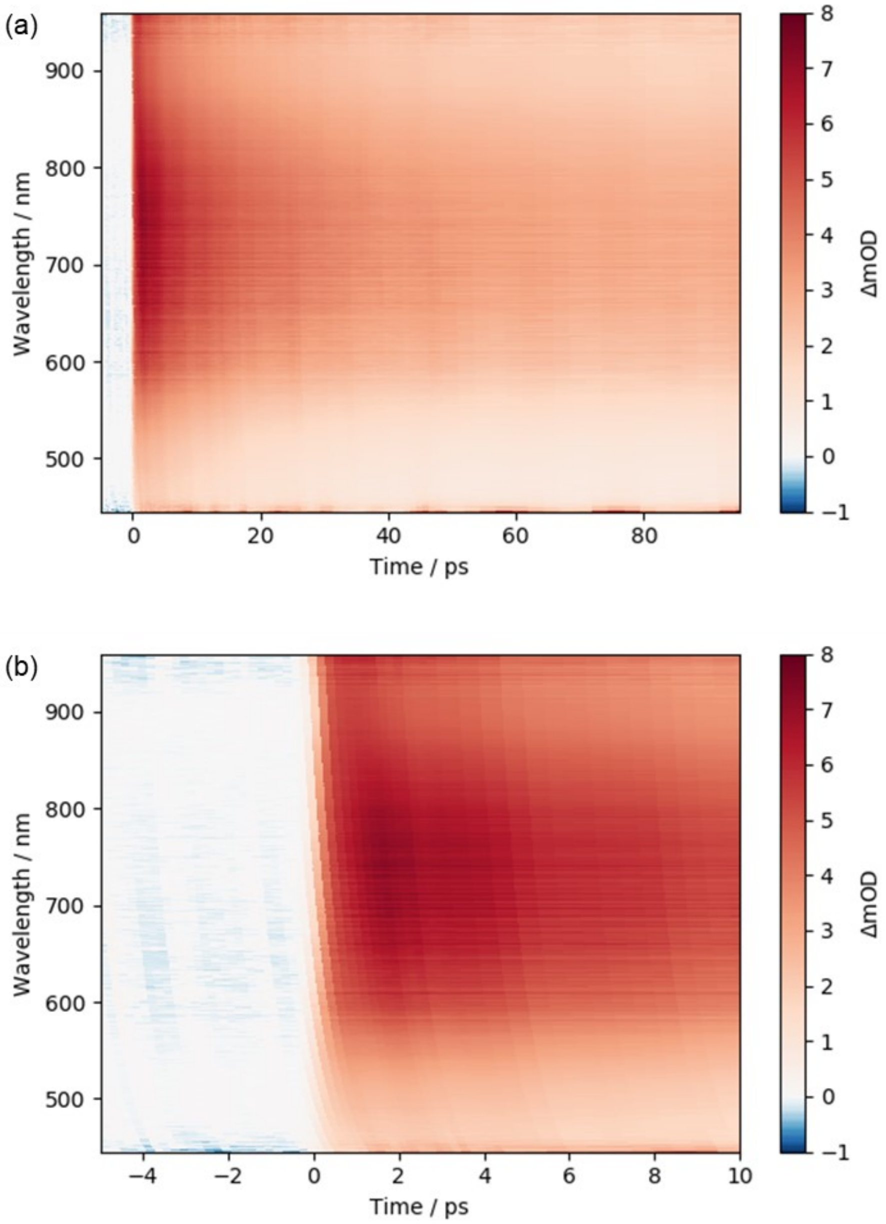

Figure S4: TA spectra of 100 mM phenolate excited at 257 nm (a) over long times, and (b) over short times.

Natural log of SFG data

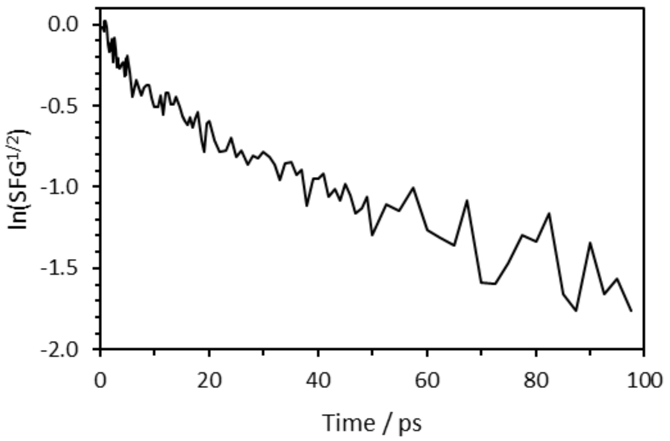

Figure S5: Natural log of data in Figure 1 of main manuscript showing that dynamics are not mono-exponential.

Errors in rate coefficients

The uncertainties in the rate coefficients were determined by an iterative method using the χ^2 (Pearson's cumulative test statistic) contour surface formed by the model fitting parameters of the proposed surface kinetic model.[6] This assumed the initial uncertainty on each data point of the SFG signal in Figure 1 was ± 0.05, estimated from the standard deviation of data points before $t = 0$. The errors quoted refer to extrapolation to the $\Delta\chi^2 = 1$ surface, corresponding to confidence limit of 1σ in each parameter.

Time-resolved SFG signal and expected hydrated electron signal in phenol

The dynamics of phenol were additionally probed following excitation at 257 nm and probing at 720 + 1028 nm. Figure S6 shows the signal obtained for phenol at three different concentrations: 20 mM, 57 mM and 200 mM corresponding to 25%, 50% and 80% surface coverage.[3] The former two concentrations were used by Tahara and coworkers in their experiments for direct comparison. Because phenol and phenolate are interconvertible by changing the pH, we additionally performed experiments on: phenolate which gave signal from $e^{-}_{(surf)}$ followed by phenol (by addition of $HCl_{(aq)}$), which gave no signal; and phenol with no signal followed by phenolate (by addition of $NaOH_{(aq)}$) to give a $e^{-}_{(surf)}$ signal.

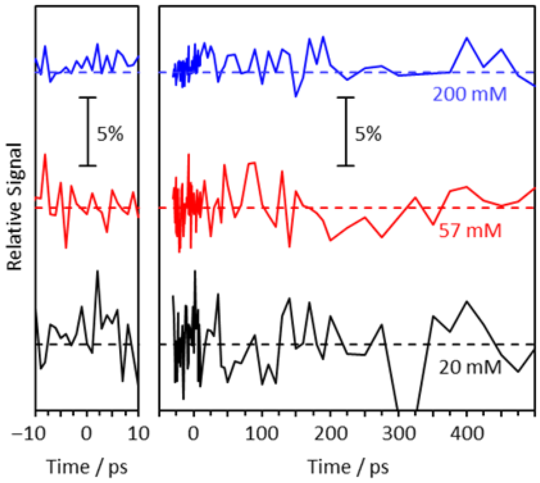

Figure S6: Raw averaged data for phenol at three different concentrations. This signal is directly comparable to the signal in Figure S3 and shows that for the different concentrations used, there is no discernible signal.

The data in Figure S6 is *directly* comparable to that in Figure S3 in terms of signal levels. That is to say, there is no discernible signal despite being sensitive to a <3% change based on the signal-to-noise in Figure S6. In contrast, Figure S3 shows that the signal levels are ~3 times larger than the (normalised) baseline. Hence, there is at least 100-times more SFG

electron signal for phenolate compared to phenol (corresponding to 10-times more hydrated electron interfacial concentration).

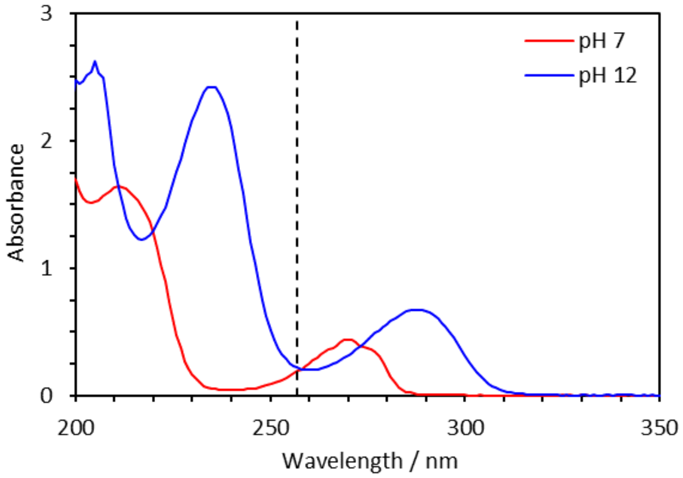

Figure S7: UV-VIS absorption spectra for 3×10^{-4} M aqueous phenol (pH 7, red) and sodium phenolate (pH 12, blue) solutions. The dashed vertical line shows the pump wavelength (257 nm) used in both TA and SFG experiments.

Some of this difference can be accounted for by the potentially larger excitation cross section for phenolate compared to phenol. Specifically, Granucci *et al* showed computationally that the second excited state (S_2) lies much closer to S_1 in phenolate than in phenol and that the S_2 has a photoexcitation cross section that is about 3 to 4 times larger than excitation to S_1.[7] At 257 nm, it is possible that the S_2 is excited rather than the S_1 state. Moreover, the cross-section of excitation of phenol at 257 nm compared to 266 nm in bulk water is approximately 3 times lower. To assess how the excitation cross sections might be affected in more detail, we have measured the absorption spectra of phenolate and phenol solutions, which are shown in Figure S7. At 257 nm, the absorption cross sections are in fact very similar, and there is insufficient difference to suggest that our experiment would not be sensitive to electrons for the phenol surface. Moreover, there is much larger concentration of molecules initially at the surface in phenol than phenolate. For phenolate, the surface coverage is ~7% (Figure S3) compared to 25%, 50% and 80% (in Figure S6) for phenol. Taking the initial concentration and the cross-

sections into account, our experiment should be sufficiently sensitive to observe the hydrated electron from photo-excited phenol at 257 nm under our experimental conditions. Note that the above discussion does not account for changes in absorption cross-section due to the interface or other factors such as initial yields of electrons, which is not known for both at the water/air interface.

Finally, we briefly explain why no fluorescence is observed for phenol compared to phenolate, even though the former has a larger quantum yield. In the experiment, a band-pass filter removes all light below 415 nm. The fluorescence spectrum of phenol does not extend to such long wavelengths, whilst that of phenolate is significantly red-shifted compared to phenol so that some fluorescence is acquired on the detector.

References

(1) Jordan, C. J. C.; Verlet, J. R. R. Time-Resolved Electronic Sum-Frequency Generation Spectroscopy with Fluorescence Suppression Using Optical Kerr Gating. *J. Chem. Phys.* **2021**, *155* (16), 164202. https://doi.org/10.1063/5.0065460.

(2) Tyson, A. L.; Verlet, J. R. R. On the Mechanism of Phenolate Photo-Oxidation in Aqueous Solution. *J. Phys. Chem. B* **2019**, *123* (10), 2373–2379. https://doi.org/10.1021/acs.jpcb.8b11766.

(3) Rao, Y.; Subir, M.; McArthur, E. A.; Turro, N. J.; Eisenthal, K. B. Organic Ions at the Air/Water Interface. *Chem. Phys. Lett.* **2009**, *477* (4–6), 241–244. https://doi.org/10.1016/j.cplett.2009.07.011.

(4) Kusaka, R.; Nihonyanagi, S.; Tahara, T. The Photochemical Reaction of Phenol Becomes Ultrafast at the Air–Water Interface. *Nat. Chem.* **2021**, *13* (4), 306–311. https://doi.org/10.1038/s41557-020-00619-5.

(5) Chen, X.; Larsen, D. S.; Bradforth, S. E.; Van Stokkum, I. H. M. Broadband Spectral Probing Revealing Ultrafast Photochemical Branching after Ultraviolet Excitation of the Aqueous Phenolate Anion. *J. Phys. Chem. A* **2011**, *115*, 3807–3819. https://doi.org/10.1021/jp107935f.

(6) Hughes, I.; Hase, T. *Measurements and Their Uncertainties: A Practical Guide to Modern Error Analysis*; Oxford University Press: Oxford: New York, 2010.

(7) Granucci, G.; Hynes, J. T.; Millié, P.; Tran-Thi, T. H. A Theoretical Investigation of Excited-State Acidity of Phenol and Cyanophenols. *J. Am. Chem. Soc.* **2000**, *122* (49), 12243–12253. https://doi.org/10.1021/ja993730j.
